# Gender-Related Differences in Sickle Cell Disease in a Pediatric Cohort: A Single-Center Retrospective Study

**DOI:** 10.3389/fmolb.2019.00140

**Published:** 2019-12-05

**Authors:** Giulia Ceglie, Margherita Di Mauro, Isabella Tarissi De Jacobis, Francesca de Gennaro, Martina Quaranta, Carlo Baronci, Alberto Villani, Giuseppe Palumbo

**Affiliations:** ^1^Department of Onco-Hematology and Cell and Gene Therapy, Scientific Institute for Research and Healthcare (IRCCS), Childrens' Hospital Bambino Gesù, Rome, Italy; ^2^University Department of Pediatrics, Bambino Gesù Children's Hospital, University of Rome Tor Vergata, Rome, Italy; ^3^Pediatric and Infectious Disease Unit, Bambino Gesù Children's Hospital, Scientific Institute for Research and Healthcare (IRCCS), Childrens' Hospital Bambino Gesù, Rome, Italy

**Keywords:** sickle-cell disease, gender medicine, pediatric anemia, hemoglobinopathies, drepanocytosis

## Abstract

Sickle cell disease (SCD) is one of the most common monogenic disease worldwide. The incidence of SCD is not strictly gender-related as it is transmitted as an autosomal recessive disorder. In particular, the gender-related differences in pediatric SCD are not well-characterized. To address this matter, we retrospectively analyzed the clinical records of 39 pediatric patients with a diagnosis of SCD (hemoglobin SS genotype) focusing on gender differences analyzing various aspects of the disease and comprising both acute symptoms and late complications. We found various gender-related differences in our pediatric population. In particular, pain crisis frequency per year was significantly increased in the male population with a mean number of crisis per year of 1.6 vs. 0.6 in the female population (*p* = 0.04). Also, severe complications (both infectious and cardiovascular) were mostly found in the male population. SCD-related late cardiac complications were observed mainly in the male population (*p* = 0.04). Our data support the hypothesis that gender could play a role in determining the clinical course of SCD, even though further studies are needed to assess the exact weight of its influence over the course of the disease. The higher morbidity in males is a well-known feature of SCD in adults and these findings have been only partially studied in the pediatric population. These differences have, in adults, been attributed to hormonal variations found in the two sexes after puberty. In a pediatric population, other factors must be responsible for these discrepancies. These findings suggest that gender could be a valuable factor in the risk stratification of these patients at diagnosis, and possibly guide therapeutic decisions, with the final aim of personalizing the therapy.

## Introduction

Sickle cell disease (SCD) is a systemic disease, associated with episodes of acute illness and progressive organ damage, and is one of the most common and severe, monogenic disorder worldwide.

The disease is caused by an aminoacidic substitution in the beta-globin gene that leads to the production of an abnormal hemoglobin called HbS. The formation of abnormal HbS is caused by a mutation in the β-globin gene in which the 17th nucleotide is changed from thymine to adenine leading to the substitution of the sixth amino-acid in the β-globin chain, glutamine acid in normal Hb becomes valine in HbS. This structural variation changes the surface of hemoglobin promoting the polymerization of hemoglobin in deoxygenation conditions. This leads to important alterations of red blood cells stability culminating in the formation of sickle-shaped erythrocytes.

Although the variation here described is the most common, the term sickle-cell disease is used to refer to different genotypes that share a similar clinical phenotype: homozygosity for the βS allele (HbS/S), heterozygosis for the βS allele and β-thalassemia (HbS/β), heterozygosity for the βS allele and some hemoglobin variants: HbS/C, HbS/D, HbS/lepore-Boston (Ware et al., 2017).

The disease mainly affects tropical regions, particularly sub-Saharan Africa, India and the Middle East, but it is also found in the Mediterranean area. The distribution of the disease in these regions is thought to be due to the so-called “Malaria hypothesis.” The hypothesis is that there is a partial resistance of HbS carriers to all forms of Plasmodium falciparum malaria so that individuals heterozygous for HbS might have had a selective advantage during malaria epidemies, thus perpetuating the mutated allele (Aidoo et al., 2002). Currently the SCD prevalence is increasing in Europe and other Western countries, mainly because of globalization and human migration. The frequency of SCD carriers in Europe is 1 subject on 150 in the general population (Piel et al., 2013).

Painful crises are the most common symptom in SCD and they are caused by recurrent acute vaso-occlusion, they usually occur whenever partially or totally deoxygenated Hb molecules polymerize, thus distorting red blood cells normal disk shape, producing stiff, sticky, sickle-shaped cells that obstruct small blood vessels and produce occlusion and consequently the disruption of oxygen to body tissues.

The incidence of sickle cell disease is not gender-related since it is transmitted as an autosomal recessive disorder. However, there have been reports of sex related differences in SCD mortality and morbidity in adult patients. For example, one study of Platt et al. showed a greater mortality in males with a mean death age 42 years for men and 48 for woman (Mortality in sickle cell disease, 2018).

Another gender dependent factor that could play a role is Nitric oxide production. Nitric oxide is thought to be important in maintaining vasomotor tone, limiting platelet aggregation, inhibiting ischemia-reperfusion injury, and modulating endothelial adhesion molecule expression (Kim-Shapiro and Gladwin, 2018). Sickle cell-related vascular phenomena of increased shear stress and compensatory responses to chronic vascular injury normally promote increased endothelial nitric oxide production, but this system is impaired in males. Estrogens facilitate nitric oxide production and limit its consumption. Since nitric oxide is linked to transcriptional control of fetal hemoglobin, it could contribute to gender differences in fetal hemoglobin expression, known to be higher in the female population. Therapies that restore nitric oxide bioactivity or reduce its consumption (or enhance non-nitric oxide induced vasodilatation) could be particularly beneficial in patients with sickle cell anemia, especially males (Gladwin et al., 2003).

Despite the presence of reports of sex related differences in SCD mortality and morbidity in adult patients, few studies (Rosenberg and Hutcheson, 2011; Kumar et al., 2018; Alexandre-Heymann et al., 2019; Amilon et al., 2019; Arigliani et al., 2019) are currently available about gender heterogeneity in the pediatric population. The objective of the present retrospective study is to find gender-related differences in the clinical course of SCD in a pediatric population.

## Methods and Materials

We conducted a retrospective review of medical records of SCD pediatric patients followed at the Bambino Gesù Childrens' Hospital in the last 12 years (2006–2018). We considered pediatric patients all subjects with <18 years of age. Patients of both sexes were diagnosed between 3 months and 11 years of age, and the data were extracted to find subjects who had a diagnosis of SCD assessed by quantitative electrophoresis and with HbS levels >45%. We excluded patients with a follow up <6 months; this decision was based on the fact that in those cases we would not have enough anamnestical and laboratory informations. The examiners conducted a retrospective chart analysis of 65 consecutively treated patients. Thirty-nine patients were identified according to the afore-mentioned inclusion and exclusion criteria.

The following parameters were analyzed:

1. Painful annual crises: Painful crises per year were recorded. Painful crises treated in other hospitals and those not needing hospitalization were excluded. Painful crises were stratified according to severity. Pain was evaluated through the visual analog scale (VAS) and divided into three category: mild (VAS: 1–3), moderate (VAS: 4–5), and severe (VAS: 6–10). Crises severity was also classified according to the therapeutical management required.
2. Treatment of painful crises:
  - Non-steroidal anti-inflammatory drugs (NSAIDs).
  - Minor opioids (tramadol or codeine).
  - Major opioids (morphine).

We considered morphine treatment in painful crises not sufficiently controlled by the previous administration of NSAIDs or minor opioids.

3. Complications:
  - Splenomegaly (and eventual splenectomy) assessed by ultrasound and defined as a spleen long axis in cm beyond the 90th percentile adjusted for age.
  - Biliary lithiasis assessed by ultrasound.
  - Heart disease assessed by echocardiographic evaluation. The main cardiac alteration correlated with SCD is ventricular eccentric hypertrophy, therefore, we considered this condition as the main cardiac complication of SCD.
  - Vascular events (Ischemic Stroke, Deep Thrombosis, Transient Ischemic Attack) evaluated by CT and MRI in symptomatic patients.
  - Osteomyelitis and septic arthritic assessed by laboratory and radiology evaluations (MRI and bone scan).
  - Number of transfusions per year.
4. Age at diagnosis: the age of the patients at the time of SCD diagnosis was recorded.
5. Laboratory evaluation of HbS, HbF.

Patient data were compiled from the records of the eligible patients using an Excel spreadsheet (Microsoft) that reflected the parameters in the patient records. Descriptive analysis was performed for numeric parameters using means and standard deviations. Parameters comparison between females and males was made by T-student, χ^2^ or Fisher test depending on the type of data analyzed and on sample size. All statistical comparisons were two-tailed and conducted at the 0.05 level of significance.

## Results

Data from 39 patients of both sexes (23 males and 16 females), with a mean age of 10.3 years (range, 3–26 years; males mean age 10.3 years and females mean age 10.5 years) followed-up for a minimum period 6 months, were collected and analyzed in the statistical analysis. Median follow-up time was 6 years (range 6 months−12 years).

A total of 89 painful crises were recorded (29 in females, 60 in males). The number of painful crises per year was calculated for each patient and means were calculated considering the sex groups (males 1.6 crisis/year, females, 0.6 crisis/year). A statistically significant difference was found between the two groups (*p* = 0.04).

As for the severity of the crisis assessed through VAS evaluation, we did not find a difference in these two groups, with 20 severe crises on 60 total crises in the male population vs. 10 severe crises on a total of 29 in the females.

In this context, we considered pain a dynamic parameter and, to evaluate it more precisely, we decided to classify the crisis according to the analgesic treatment required. The percentages related to the treatment of crises are described in the table below (Table 2) and a greater use of morphine was observed in males than females (*p*-value = 0.008).

Cardiopathy: 31 out of the 39 patients underwent cardiac assessment. Cardiac anomalies were detected in 13 patients, including 10 males (77%) and 3 females (23%). A statistically significant difference was found between the two groups (*p* = 0.04).

Splenectomy: Splenectomy interventions were performed in two patients of the group of males (9%) and in two patients of the group of females (19%).

Splenomegaly: Patients who performed at least one abdominal ultrasound were 38 (22 males and 16 females). Splenomegaly was found in 12 patients in the males group (55%) and in 10 patients in the females group (63%).

Biliary lithiasis: Cholelithiasis was found in two male children (14%) and six females (38%) of the group. Cholecystectomy interventions were recorded in two female patients (13%).

Vascular events: A case of transient ischemic attack (TIA) and an episode of venous portal vein thrombosis have been observed, both of which in male patients (8.7%).

Osteomyelitis and septic arthritis: Four cases of osteomyelitis have been reported, of which three were found in male (14%) and one in a female (6%).

Transfusions: The analysis of the median number of transfusions per year was almost identical in the two groups: a mean of 2.3 transfusions/year was reported for males and 1.9 transfusions/year for females.

The age at diagnosis was assessed for all patients followed by the OPBG Center, from 2006 onwards. Patients whose SCD diagnosis was made elsewhere then OPBG were not included in this analysis. The group included 30 patients (15 males, 15 females). The results of this comparison showed that the average age at diagnosis in the group of males was 1.7 years, while in the group of females 4.3 years (Table 3) (see Tables 1 and 3 for a summary of the parameters evaluated).

**Table 1:** Descriptive analysis of the complications recorded.

	**Males**	**Females**
Splenomegaly	12 (55%)	10 (63%)
Splenectomy	2 (9%)	3 (19%)
Biliary lithiasis	3 (14%)	6 (38%)
Cholecystectomy	0	2 (13%)
Cardiopathy	10 (77%)	3 (23%)
Vascular events	2 (9%)	0
Osteomyelitis	3 (14%)	1 (6%)
Transfusions/year	2.3	1.9

**Table 2:** Analysis of the different pain treatment in males and females.

	**Males**	**Females**	***p*-value**
Paracetamol	50%	55%	0.79
Ibuprofen	2%	3%	0.6
Toradol	33%	23%	0.37
Tramadol	37%	38%	0.93
Morphine	33%	7%	0.008

**Table 3:** Descriptive analysis of the age at diagnosis laboratory evaluation of HbS, HbF, and hemolytic state (LDH and bilirubin).

	**Males**	**Females**	***p*-value**
Age at diagnosis	1.7 years	4.3 years	0.88
HbS	64.27%	65.94%	0.6
HbF	10.46%	12.78%	0.25
LDH	948.7 UI/L	939.4 UI/L	0.92
Bilirubin	2.32 mg/dl	2.61 mg/dl	0.64

As for HbS level, we found a median value of 62.47% in the males vs. a 65.94% in the females. This difference was not statistically relevant: *p* = 0.6. Fetal hemoglobin values were found to be slightly more elevated in the females group. The median value for the males was in fact 10.5% while in the females it was 12.8%, *p* = 0.25 No alpha globin mutations were found in our population.

## Discussion

Sickle cell anemia is a monogenic disease but presents a very complex phenotype and very variable clinical manifestations between subjects. In this study, conducted on 39 patients followed at Bambino Gesù Childrens' Hospital, we analyzed the main factors characterizing the pathology to assess if gender could have an influence in the clinical and therapeutic course of the disease.

To the best of our knowledge scarce data are available in the literature about SCD sex differences in pediatric population. Sex hormones were recognized as responsible for gender differences in adult patients with SCD, but in the pre-puberty setting of childhood their role could be less relevant in the pathogenesis of gender differences in the pediatric population (Gladwin et al., 2003; Kato et al., 2007; Nebor et al., 2011; Jit et al., 2019).

The aim of this study was to find gender-related differences in the clinical course of SCD in a pediatric population.

Our first focus concerns the number of painful crises per year; painful crises, in fact, in addition to being very heterogeneous, are also highly unpredictable and the few studies in literature addressing this issue speculate that the reduction of fetal hemoglobin, the increase of hematocrit and leukocyte counts, are, among the parameters studied, the only ones associated with a higher rate of crisis (Niscola et al., 2009). Moreover, an annual average of crisis between the 0.4 and the 0.8 per patient is reported, regardless of the sex of patients (Kato et al., 2007; Niscola et al., 2009; Nebor et al., 2011; Jit et al., 2019).

Our results showed that males had more episodes of painful crises per year than females (*p* = 0.04), with an average higher than that reported in the literature. The reason of that difference could be attributed to the different bioavailability of nitric oxide, higher in females, as suggested in various studies (Gladwin et al., 2003; Ilesanmi, 2010).

Concerning SCD clinical complications, the literature shows a greater severity of clinical manifestations in males (Lamarre et al., 2013).

In the present study a high variability in the results was noticed: the comparison between the incidence of splenomegaly in the two groups showed no gender specificity, whereas the incidence of cholelithiasis tended to be more frequent in females, as extensively documented in literature (Currò et al., 2007).

In the category of cardiopathies, eccentric left ventricular hypertrophy is the most frequently reported in SCD patients. The present study showed that males are most frequently affected by this complication (*p* = 0.04). Same results were already obtained by Morrison et al. at the Lady's Children's Hospital Crumlin (Morrison et al., 2018).

Concerning the incidence of vascular events, a total of two episodes was recorded (5%), both cases in male patients. Osteomyelitis also manifested mainly in males (three episodes in males and 1 in females) confirming the tendency that males are more prone to complications than females.

Transfusions number was also analyzed. The initial hypothesis was that males needed more transfusions, as transfusions improve oxygenation and disrupt the intravascular mowing process by dilution (in the case of simple transfusion) of pathological red blood cells containing HbS. However, the results did not support this hypothesis, showing an identical average between males and females.

In our sample males were more represented than females. The sample size of male patients and the fact that they had more episodes of painful/annual crises, has led us to analyze the average age to the diagnosis of the two groups: males would have an earlier diagnosis, as they have a worse clinical course than females and they need a greater number of hospitalizations and investigations. This could be a possible explanation to the higher number of males in our pediatric sample compared to adult populations, in which the sample is more gender balanced. The results of this analysis confirmed our theory, for males the average age is 2 years, for females 4 years.

Regarding vaso-occlusive crises an analysis of the treatment necessary to control the pain during painful crises was performed. The pain, in the crises, is classified according to the visual analog scale (VAS) scale in mild, moderate and severe and the most recent AIEOP (Italian Association of Hematology Pediatric Oncology) guidelines recommend the use of morphine in the pain control of moderate-severe crises. The analysis of our data showed that, the percentage of severe painful crises (VAS > 6) is very similar in the two groups and, nevertheless, almost exclusively male children have needed a treatment with morphine, with “*p*” < 0.05. Also, this data confirms our hypothesis: males seem to have a worse clinical course than females, thus requiring more important pain therapy than females. Many studies in literature described gender differences in frequency and intensity of pain. In these studies, women often report lower pain thresholds, higher pain ratings, and lower tolerance for pain (Sorge and Totsch, 2017). Nevertheless, these assumptions have not been reported in SCD patients both in adult (McClish et al., 2006) and in pediatric populations (Fosdal, 2015). Since these patients experience both chronic and acute pain, there might be a long-term modulation of pain sensitivity. Any differences in gender in this regard should be more thoroughly assessed in further studies.

As for laboratory results, we didn't find any statistically relevant difference in the two groups, the slightly higher median value for HbF in females has already been described in adults (Amid and Odame, 2014).

In conclusion, our results confirmed that gender plays a role in the pathogenesis and in the course of the disease, in particular, male gender seems to represent an indicator of a more aggressive disease course. Thus, our results showed that there is more morbidity in the male sex. This data had not yet been directly addressed in any study in the pediatric age, although it has already been shown in the adult population. The gender-specific differences observed, partly already known in the adult, have always been attributed to the hormonal changes that are physiologically present in the two sexes after puberty. However, in the pediatric population, other factors must be implicated in determining the described differences. Further studies are encouraged to highlight possible risk factors connected to gender in the SCD pediatric population. Therefore, taking these preliminary data, and their possible confirmation in wider studies, male gender could be taken into account in the initial assessment of the patients. This could, in fact, represent a simple and intuitive risk factor that could be implemented in the prognostic stratification since diagnosis, thus leading to personalized therapeutic decisions for the two sexes and the implementation of major prevention and surveillance programs for males.

## Data Availability Statement

The datasets generated for this study are available on request to the corresponding author.

## Author Contributions

GC, MD, and GP designed the study. GC, MD, MQ, FG, and CB cured the collection of the data. GC, MD, IT, FG, AV, and GP interpreted and analyzed the data. GC and MD drafted the manuscript. AV and GP critically revised the manuscript for intellectual content.

### Conflict of Interest

The authors declare that the research was conducted in the absence of any commercial or financial relationships that could be construed as a potential conflict of interest.
